# Dementia and Risk Factors: Results from a Prospective, Population-Based Cohort Study

**DOI:** 10.3390/life12071055

**Published:** 2022-07-14

**Authors:** Simona Villani, Ottavia Eleonora Ferraro, Tino Emanuele Poloni, Antonio Guaita

**Affiliations:** 1 Unit of Biostatistics and Clinical Epidemiology, Department of Public Health, Experimental and Forensic Medicine, University of Pavia, Via Forlanini 2, 27100 Pavia, Italy; 2 Golgi Cenci Foundation, 20081 Abbiategrasso, Italy; e.poloni@golgicenci.it (T.E.P.); a.guaita@golgicenci.it (A.G.)

**Keywords:** dementia, walking speed, comorbidity

## Abstract

The incidence rate of dementia varies between studies. The influence of some sociodemographic factors is reasonably established, but less is known about the role of comorbidities, which are common in the elderly. The objectives of this study was to estimate the incidence of dementia in a population of Italian elders and evaluate the role of walking speed, comorbidity and ApoE-ɛ4 as well as various sociodemographic factors on the new onset of dementia. The InveCe.Ab study is a population-based longitudinal study in people born between 1935 and 1939 and resident in Abbiategrasso, Milan, Italy. After excluding subjects with a diagnosis of dementia and those without a definite diagnosis, 1103 individuals with a median follow-up time of 4.1 years were included in the analyses. The cumulative four-year incidence of dementia was 5.3%. Demographic factors such as old age, male, less educated, ApoE-ɛ4 carrier status and slower gait were risk factors for dementia onset in a cognitively healthy sub-cohort. Comorbidity did not influence the onset of dementia; instead, slow walking speed appears to be a strong predictor of dementia onset.

## 1. Introduction

The number of dementia cases reported in published studies varies widely [1]. A meta-analysis that included 10 studies estimated an approximately 52.9 per 1000 persons pooled annual incidence of dementia in the over-65 population [2]. However, the studies included in the meta-analysis showed a pooled incidence that varied from 8.7 per 1000 person-years in a Japanese study [3] to 142.2 per 1000 person-years in a U.S. study [4].

Several years ago in Italy, two studies on dementia were performed. The ILSA study, carried out more than 20 years ago [5], estimated an incidence rate of dementia equal to 12.5 per 1000 person-years, while the Conselice study, conducted more 10 years ago [6], reported a very different incidence rate: 37.8 per 1000 persons-years.

Among the sociodemographic factors that may influence the incidence of dementia, age is the one known to show the strongest association in any world area [1], while the role of gender is still uncertain [7]; many studies have failed to detect a gender difference in overall dementia rates [2,5,6,8,9,10,11,12,13,14,15,16,17]. A higher level of education seems to have a clear inverse relationship with dementia onset, exerting a protective effect [18]. Other clinically related factors, such as comorbidities, have been less investigated and need to be verified as possible independent predictors of dementia onset (i.e., walking speed). Comorbidity is an issue that tends to be neglected in dementia studies [19], but the walking test is considered to be a marker of health and fitness in aging [20].

Among genetic backgrounds, the ApoE-ɛ4 allele has long been established as the principal genetic risk factor for dementia, mainly Alzheimer’s disease [21] but also other forms of non-vascular dementia in both homozygous and heterozygous carriers [22].

Moreover, aging and dementia risk is a complex phenomenon depending on other risk factors related to life experience, as recently indicated by Martinez-Escudero et al. and Gimenez-Serrano et al. [23,24]

The primary aim of this research was to estimate the incidence of dementia in a population of either cognitive healthy or mildly impaired elders at baseline who were living in a homogeneous urban area in the hinterland of Milan, Italy. Its secondary aim was to ascertain the predictive roles of walking speed, comorbidities, ApoE-ɛ4 and sociodemographic factors, specifically year of birth, education and gender on the onset of dementia.

## 2. Materials and Methods

### 2.1. Study Design, Participants and Setting

The InveCe.Ab (“Invecchiamento Cerebrale in Abbiategrasso” or “Brain aging in Abbiategrasso”) study (ClinicalTrials.gov, NCT01345110) is an ongoing longitudinal population-based study involving all elderly people born between 1935 and 1939 and living in Abbiategrasso, a town with 32,000 inhabitants situated near Milan in northern Italy. The InveCe.Ab study methodology has been described in detail elsewhere [25]. Briefly, all selected members of the selected birth cohort officially residing in Abbiategrasso in November 2009 formed the InveCe.Ab population and were invited to undergo a multidimensional assessment (social, medical and neuropsychological) and provide a blood sample for biological and genetic analyses. All the InveCe.Ab study participants gave their written informed consent to the study, whose procedures were conducted in accordance with the Declaration of Helsinki. The study protocol was approved by the Ethics Committee of the University of Pavia.

Of the 1644 individuals eligible to participate in the baseline assessment in 2010, 1321 (80.4%) agreed to take part in the study. Of the 1321, 1267 participants were undiagnosed with dementia. Only 39 subjects had received a diagnosis of dementia, and 15 participants had inconclusive results. All of the respondents were invited to undergo a follow-up assessment in 2012, and 1114 of the 1245 individuals eligible at this stage (89.5%) were re-examined using the same methodology adopted for the baseline assessment. In 2014, all individuals who were still living and who had participated in either the baseline and first follow-up assessment or only in the baseline assessment were invited to attend a second follow-up. Those still eligible numbered 1061, and 988 of them accepted the invitation.

Only subjects who belonged to the at-risk cohort (i.e., the 1267 respondents without a dementia diagnosis) and had attended at least one follow-up visit after the screening phase were considered for the present study; accordingly, 1103 subjects were included in the analysis. Of these, 1021 were cognitively healthy, and 82 were cognitively impaired, as defined in a previous paper [26]: 52 had mild cognitive impairment (MCI), and 30 had cognitive impairment, no dementia (CIND).

### 2.2. Endpoint

The primary endpoint was new onset of dementia during the follow-up period. As in the previous prevalence study [27], dementia was diagnosed according to the Italian version of the *Diagnostic and Statistical Manual of Mental Disorders IV* (DSM IV-TR) [27]. An individual was considered affected by dementia when agreement on this diagnosis was reached between a psychologist and a geriatrician. If the physicians failed to agree, the diagnosis had to be confirmed by a second geriatrician.

### 2.3. Selected Variables

In detail, the following variables were considered:

- Birth cohort: 1938–1939 vs. 1935–1937.
- Gender: male, female.
- Years of education: dichotomised as over 3 years (>3 years, reference category) or up to 3 years (≤3 years).
- Walking Speed Test (WST): time, in seconds, taken to walk back and forth along a special five-metre pathway without pausing. This was part of a Talking While Walking Test [28]
- Comorbidity index from the Cumulative Illness Rating Scale: The index, ranging from 0 to 12 points, represents the number of body areas with active disease [29] excluding the psychiatric area; the higher the comorbidity score, the worse the subject’s health.
- ApoE-ɛ4 allele: The presence of the ApoE-ε4 allele was ascertained by means of real-time PCR. Genomic DNA was extracted from blood samples using an automated nucleic acid extraction system (Maxwell; Promega, Madison, WI, USA). Genotyping analysis was conducted by real-time polymerase chain reaction (PCR) allelic discrimination using TaqMan^®^ probes in a CFX 384 real-time PCR system (Bio-Rad, Hercules, CA, USA) and available pre-made assays (Applied Biosystems, Foster City, CA, USA). APOE common variants (ε2, ε3, ε4) were determined from the combination of two SNPs, rs7412 and rs429358, within codons 112 and 158 of the APOE gene, respectively (C_904973 and C_3084793 assays for rs7412 and rs429358, respectively. Allele calling was based on the clustering algorithm implemented in CFX Manager™ software, version 3.1 (Bio-Rad, USA). APOE genotype information was simplified to a binary classification based on the absence or presence of at least one ɛ4 allele.

### 2.4. Statistical Methods

The quantitative variables were summarised as means with standard deviation and as minimum and maximum values. For the qualitative variables, absolute frequencies and percentages were reported. To evaluate differences in quantitative variables between the sub-cohorts within the population at risk of new-onset dementia (the cognitively healthy and the cognitively impaired), an unpaired *t*-test (with Satterthwaite’s degrees of freedom correction if necessary) was used. Either a chi-square test or Fisher’s exact test was applied to describe the associations of the qualitative variables both with cognitive status at baseline and with the occurrence of dementia during the course of the follow-up.

The crude cumulative rate (or risk) of dementia was calculated, and risk ratios (RRs) with 95% confidence intervals (95%CIs) were estimated. A log-binomial model or the modified Poisson approach using robust error variances was applied [30] to estimate the crude and adjusted associations of each potential factor investigated in bivariate analysis. These models were applied only to the cognitive healthy sub-cohort with complete information on all the variables. A *p*-value of less than 0.05, two-tailed, was considered significant. Analyses were conducted using STATA version 15 (StataCorp. 2017. Stata Statistical Software: Release 15. StataCorp LLC., College Station, TX, USA).

## 3. Results

The 1103 members of the at-risk cohort considered in the present study had a larger percentage of females and a mean age of 78.5 years (±1.4 years; range 72.6–79.8 years) at the end of four years of follow-up. Subjects born in 1935–1937 were slightly more numerous than those born in 1938–1939 (Table 1). Around 8% of the cohort reported less than three years of schooling. Almost a fifth carried the ApoE-ɛ4 allele. After analysing the cohort by baseline cognitive status, it was found that the cognitively healthy subjects had a considerably higher proportion of females compared with the cognitively impaired subjects, but no other characteristic differed between these two sub-cohorts (Table 1).

### 3.1. New Onset of Dementia

The cumulative incidence of dementia during the entire follow-up period was 5.3%, significantly higher in those with cognitive impairment at baseline (Table 2).

The cumulative incidence of dementia did not differ by gender (Table 3). Participants from the 1935–1937 birth cohort were three times more likely than the younger cohort to develop dementia; this increased risk was statistically relevant. Education also emerged as a relevant factor influencing new onset of dementia, with low education significantly increasing the risk (Table 3). The cumulative incidence of dementia was twice as high in the ApoE-ɛ4 allele carriers than in the non-carriers, but this was not statistically significant. Gait speed was found to be associated with a significant increase in the risk of new onset of dementia, with the subjects who developed dementia recording, on average, a longer walking test time. The comorbidity score showed no relevant association.

### 3.2. Factors Associated with New Onset of Dementia

The multivariate analysis confirmed the predictive role of the walking test: a longer walking test time at baseline significantly increased the risk of developing dementia independent of the other factors (Table 4). On the contrary, there was no confirmed association with the onset of dementia after adjusting for the other factors. Moreover, male gender, older age and ApoE-ɛ4 carrier status were independent predictors of the development of dementia in the course of the follow-up period (Table 4).

The association with potential risk factors was investigated separately for males and females (Table 4). Females showed a significantly increased risk of dementia in those with a slower walking speed and a low education level. Among males, slower walking speed was a significant independent predictor of new-onset dementia. Contrary to expectations, comorbidities showed a “protective” role in that a greater number of comorbidities lowers the risk of developing dementia.

## 4. Discussion

The main findings of this study can be summarised in the following points:

- The risk of new-onset dementia over the four-year follow-up period in the cohort of at-risk elders is around 5%.
- The risk of developing dementia is around eight times higher in people with cognitive impairment at baseline with respect to the cognitively healthy.
- The risk of new onset of dementia is greater in elders with slower walking speed at baseline, in ApoE-ɛ4 carriers, in males and in older individuals; education and comorbidity (measured by CIRS index) show no independent relationship with dementia incidence.
- Elderly females and males showed different risk profiles of dementia; in females, a slower walking speed and low education are important prognostic factors associated with the onset of dementia. In males, only the walking speed at baseline and comorbidity are significantly related to the risk of dementia.

### 4.1. Four-Year Cumulative Dementia Incidence

The reported overall risk of new-onset dementia of 5.3 is similar to that estimated in a cohort incidence study in the context of the Italian Longitudinal Study on Aging (ILSA) [6]; it is also in line with findings of many studies from other countries. However, it differs from the Conselice study findings [6]. Elders showing cognitive impairment at baseline in the InveCe.Ab study have a higher risk of developing dementia compared with their cognitively healthy peers (26.8% vs. 3.5%).

The rate of progression to dementia found in the cognitively impaired members of the present study cohort is considerably lower than that reported in other studies (about 10% per year but showing considerable variability) [31,32], a difference that may depend on the source of the participants recruited: cognitively impaired participants referred from memory clinics or similar diagnostic centres showed a much higher rate of progression than those drawn from population studies. A study comparing the progression of MCI to dementia in a clinic versus community-based cohorts reports rates of 13% versus 3%, respectively [33]. Similarly, ILSA shows a 3.8% rate of progression to dementia from MCI [34]. Therefore, the InveCe.Ab findings are in line with those reported in similar population studies.

The variability existing between the present findings and those of some previous research could be due to differences in the diagnostic criteria and assessment instruments used, in the compositions of the populations or in the choice of study design (dual- or single-step assessment methodology). In most previous studies, participants underwent cognitive screening before being accepted for neuropsychological and medical evaluation; this may have introduced a diagnostic bias, especially regarding mild dementia. The InveCe.Ab study, based on the comprehensive assessment of all participants, avoided this issue.

### 4.2. Relationship between New Onset of Dementia and Predictors

#### 4.2.1. Gait Speed

The finding that WST performance shows an inverse relationship with the incidence of dementia that was significant in both men and women confirmed that the WST could be prognostic for developing dementia. The present findings are in agreement with primary literature. Several studies have examined the longitudinal relationship between gait speed and cognition and concluded that gait speed is a good predictor of both general [35,36,37,38,39] and specific cognitive function decline [40,41]. Gait speed could be an independent predictor of dementia onset in elderly people [35,42].

#### 4.2.2. ApoE-ɛ4, Gender, Age, Education and Comorbidity

The results of the present study confirm the strong influence of ApoE-ɛ4 status on dementia onset that has previously been demonstrated by others [21,43].

The absence of differences in dementia risk by gender is consistent with findings from previous studies [6,10,11,12,14,15]. In a 2014 review on a sex–gender influence on the incidence of dementia and Alzheimer’s disease [44], while the authors found that the majority of studies had not observed sex differences in the rates of developing AD, ten studies (from the USA, Europe, and Asia) reported a higher incidence of AD in women. However, in all studies, this difference emerged after 80 and especially 85 years of age: Studying 16,926 women and men in the Swedish Twin Registry aged 65+, the authors found a higher dementia rate for women older than 85 years and a higher Alzheimer’s disease rate for those older than 80 years [44]. The same results had been found from previously analysing gender and incidence of dementia in the Framingham Heart Study [10]. Therefore, we can reasonably conclude that we should expect to find gender differences in dementia incidence rate in ages older than those of our population.

As universally found in the related literature, age is an important risk factor for new onset of dementia in the elderly population. In this InveCe.Ab-based study, the incidence of new-onset dementia is lower in the younger birth cohort, even though this finding must be set within the context of the narrow age range of the overall study population (less than five years).

The study also shows that a higher education level (more than 3 years) exerts a protective effect on dementia incidence at least in the female gender, in agreement with two previous Italian studies that also applied for education a 3-year cut-off [6,34] and in line with the position paper on sex and gender differences in dementia by Ferretti et al. [45]. The protective effect of education is also reported in two meta-analyses [18,46] and in line with the cognitive reserve hypothesis [47].

In contrast with previous data on high comorbidity in individuals with dementia, we found that comorbidities are not predictors of dementia cases [19,48]. However, these articles reported a simple association between dementia and comorbidity but did not analyse comorbidities as risk factors for the onset of dementia. These findings converge to indicate that comorbidity is an accompanying factor in dementia syndrome and may influence its clinical evolution [49] but is not in itself a short-term risk factor.

### 4.3. Strengths and Limitations

The cohort study design used in this research allowed for the estimation of the four-year cumulative incidence of new-onset dementia in a population of elderly people living in a homogeneous urban area, monitored by the same staff and evaluated using the same methodology. This approach minimized the risk of information bias. A second strength of the study is that each participant underwent a complete single-step multidimensional assessment, as opposed to the two-step assessment used in most previous studies in this field. Third, the good response rate achieved at each of the scheduled follow-up assessments avoided the risk of a possible selection bias. Nevertheless, the study has some limitations.

First, the narrow age range of the participants (70–74 years) limits the generalizability of the conclusions, although it helped to avoid a selection bias and made it possible to target an interesting life stage, that of the transition from late adulthood to old age [26]. Second, all participants are living in a definite small area of a developed country and are Caucasian, so the results cannot be applied to populations with different characteristics. The third limitation concerns the diagnostic process, to which less time could be devoted than would be the case in a clinical setting. To overcome this weakness, each diagnosis was reached by agreement between a geriatrician and a psychologist, and if necessary (in case of lack of agreement or uncertainty) confirmed by another independent geriatrician. Finally, other risk factors not collected and related to cumulative lifespan experience might add knowledge and enrich the complex set of factors that could help to explain the onset of dementia.

## 5. Conclusions

The results of the present study confirm previous literature evidence on the influence of certain factors, such as age walking speed, education and ApoE-ɛ4 status on the incidence of dementia. The particular importance of gait speed, though it is less considered in previous research on dementia incidence, lies in the fact that it can be targeted in prevention programs, but further studies are needed to verify if increasing walking speed might reduce the onset of dementia. Future studies exploring ApoE-ɛ4 patterns might also confirm the possible role of these aspects in the prevention of dementia. Finally, the role of comorbidity or multimorbidity in the mechanism related to cognitive impairment and the subsequent development of dementia should be further explored in new longitudinal studies using different tools.

## Figures and Tables

**Table 1:** Characteristics of the at-risk cohort at baseline composed of cognitively healthy and cognitively impaired elders.

	*At-Risk* *Cohort*		Cognitively Healthy		Cognitively Impaired		Test and *p*-Value
	*n = 1103*		*n* = 1021		*n* = 82		
	*n*	*%*	*n*	%	*n*	%	
Females	*593*	*53.8*	569	55.7	24	29.3	Chi^2^ = 21.38; *p* < 0.001
1935–1937 birth cohort	*590*	*53.5*	540	52.9	50	61	Chi^2^ = 2.00; *p* = 0.158
Up to 3 years of education	*83*	*7.5*	77	7.5	6	7.3	Chi^2^ = 0.006; *p* = 0.941
ApoE-ɛ4	*201*	*18.3*	183	18	18	22	Chi^2^ = 0.83; *p* = 0.363
	*Mean*	*SD*	*Mean*	*SD*	*Mean*	*SD*	**Test and** ***p*-value**
Walking test (minutes)	*13.6*	*2.9*	13.5	2.8	14	2.9	t = 1.50; *p* = 0.134
Comorbidity index score	*2.2*	*1.4*	2.2	1.4	2.3	1.5	t = 0.66, *p* = 0.512

**Table 2:** Cumulative incidence of dementia (D) during the 4-year follow-up (2010–2014).

	*At-Risk Cohort*	Cognitively Healthy	Cognitively Impaired	*p*-Value
	*n = 1103*	*n* = 1021	*n* = 82	
New onset of dementia	*5.3*	3.5	26.8	<0.0001

**Table 3:** The crude associations (risk ratios, RRs) with 95% confidence intervals (95%CI) of personal and behavioural characteristics with the onset of dementia in the cognitively healthy cohort ^§^.

	RR	95%CI		*p*-Value
**Gender**				
Females vs. Males	0.99	0.49	1.99	0.983
**Birth cohort**				
1935–1937 vs. 1938–1939	3.12	1.36	7.14	0.007
**Years of education**				
Up to 3 years vs. Over 3 years	3.10	1.31	7.31	0.010
**ApoE-ɛ4**				
Carriers vs. no Carrriers	1.87	0.87	3.99	0.106
**Walking test time**	1.22	1.20	1.24	<0.001
**Comorbidity**	1.17	0.91	1.49	0.219

^§^ 974 were the cognitively healthy with no missing data. Log-binomial models using robust error variances were applied.

**Table 4:** Factors associated with new onset of dementia during the follow-up period in the cognitively health cohort with no missing data ^§^.

	At-Risk Cohort				Male				Female			
	*n* = 974				*n* = 438				*n* = 536			
	RR	[95%CI]		*p*-Value	RR	[95%CI]		*p*-Value	RR	[95%CI]		*p*-Value
**Gender**												
Males	1											
Females	0.46	0.22	0.96	0.040								
**Birth cohort**												
1938–1939	1				1				1			
1935–1937	2.42	1.05	5.58	0.039	1.64	0.52	5.13	0.395	2.76	0.77	9.92	0.120
**Years of education**												
Over 3 years	1				-				1			
Up to 3 years	2.00	0.88	4.56	0.098					2.74	1.02	7.38	0.046
**ApoE-** **ɛ4 allele**												
Non-carriers	1				1				1			
Carriers	2.14	1.02	4.5	0.044	1.53	0.46	5.08	0.487	2.53	0.96	6.64	0.06
**Walking test time**	1.24	1.19	1.31	<0.001	1.44	1.32	1.57	<0.001	1.22	1.14	1.31	<0.001
**Comorbidity**	0.9	1.19	1.31	0.443	0.95	0.94	0.96	<0.001	0.87	0.57	1.33	0.511

^§^ A log-binomial model using robust error variances was applied.

## Data Availability

The data are available on request.
